# Novel and de novo point and large microdeletion mutation in *PRRT2*‐related epilepsy

**DOI:** 10.1002/brb3.1597

**Published:** 2020-03-31

**Authors:** Li Yang, Cuiping You, Shiyan Qiu, Xiaofan Yang, Yufen Li, Feng Liu, Dongqing Zhang, Yue Niu, Liyun Xu, Na Xu, Xia Li, Fang Luo, Junli Yang, Baomin Li

**Affiliations:** ^1^ Department of Pediatrics Qilu Hospital of Shandong University Jinan China; ^2^ Department of Pediatrics Linyi People’s Hospital Affiliated to Shandong University Linyi China; ^3^ Central Laboratory Linyi People’s Hospital Affiliated to Shandong University Linyi China; ^4^ Department of Neurology Zibo Zhangdian Hospital of Traditional Chinese Medicine Zibo China; ^5^ Department of Pediatrics Shandong medical college Linyi China; ^6^ MyGenostics Inc Beijing China

**Keywords:** 16p11.2 deletion, copy number variants, epilepsy, myoclonic seizures, *PRRT2*

## Abstract

**Background:**

Point and copy number variant mutations in the *PRRT2* gene have been identified in a variety of paroxysmal disorders and different types of epilepsy. In this study, we analyzed the phenotypes and *PRRT2*‐related mutations in Chinese epilepsy children.

**Methods:**

A total of 492 children with epilepsy were analyzed by whole exome sequencing (WES) and low‐coverage massively parallel CNV sequencing (CNV‐seq) to find the single nucleotide variants and copy number variations (CNVs). And quantitative polymerase chain reaction was utilized to verify the CNVs. Their clinical information was followed up.

**Results:**

We found *PRRT2*‐related mutations in 19 patients (10 males and nine females, six sporadic cases and 13 family cases). Twelve point mutations, four whole gene deletion, and three 16p11.2 deletions were detected. The clinical features of 39 patients in 19 families included one early childhood myoclonic epilepsy (ECME), one febrile seizure (FS), two infantile convulsions with paroxysmal choreoathetosis (ICCA), six paroxysmal kinesigenic dyskinesias (PKD), 12 benign infantile epilepsy (BIE), and 17 benign familial infantile epilepsy (BFIE). All patients had normal brain MRI. Interictal EEG showed only one patient had generalized polyspike wave and five patients had focal transient discharges. Focal seizures originating in the frontal region were recorded in one patient, two from the temporal region, and two from the occipital region. Most patients were treated effectively with VPA or OXC, and the child with myoclonic seizures was not sensitive to antiepileptic drugs.

**Conclusion:**

*PRRT2* mutations can be inherited or de novo, mainly inherited. The clinical spectrum of *PRRT2* mutation includes BIE, BFIE, ICCA, PKD, FS, and ECME. The *PRRT2*‐related mutations contained point mutation, whole gene deletion and 16p11.2 deletions, and large microdeletion mutations mostly de novo. It is the first report of *PRRT2* mutation found in ECME. Our report expands the mutation and clinical spectrum of *PRRT2*‐related epilepsy.

## Graphical abstract

*PRRT2* mutations can be inherited or de novo, mainly inherited. The clinical spectrum of PRRT2 mutation includes BIE, BFIE, ICCA, PKD, FS, and ECME. The PRRT2‐related mutations contained point mutation, whole gene deletion and 16p11.2 deletions, and large microdeletion mutation mostly de novo. Our report expands the mutation and clinical spectrum of *PRRT2*‐related epilepsy.

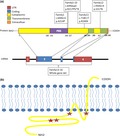

## 1 INTRODUCTION

Mutations in the proline‐rich transmembrane protein 2 (*PRRT2*) gene on chromosome 16 were first identified in the paroxysmal kinesigenic dyskinesias (PKD) family and subsequently in benign familial infantile epilepsy (BFIE) and infantile convulsions with paroxysmal choreoathetosis (ICCA) (Mathot, Lederer, Gerard, Gueulette, & Deprez, 2017; Okumura et al., 2019; Zhao et al., 2019) and were also reported to be associated with benign infants myoclonic (Maini et al., 2016), west syndrome (Djemie et al., 2014), febrile convulsions (FS) (Zheng et al., 2016), hemiplegic migraines (Cloarec et al., 2012), intermittent ataxia, and other differential movement disorders (Castelnovo et al., 2016; Delcourt et al., 2015; Ebrahimi‐Fakhari et al., 2014; Legris et al., 2019). Homozygous *PRRT2* mutations give rise to the more severe clinical disease of mental retardation and intermittent ataxia (Labate et al., 2012). All of these findings broaden the phenotype caused by *PRRT2* mutations, from which a definition of *PRRT2*‐related diseases emerged. The phenotypes of *PRRT2*‐related diseases were incompletely extraneous, and the affected members of the same family had phenotypic heterogeneity (Cloarec et al., 2012).

Nonsense mutations containing premature termination codon (PTC) are the most common type, and c.649dupc (p.r217pfsx8) is a hot spot mutation (Fabbri et al., 2013; Zhang, Li, Chen, Gan, & Liu, 2017). Copy number deletions of this gene such as 16p11.2 deletion are also known to cause *PRRT2*‐related diseases (Dale, Grattan‐Smith, Nicholson, & Peters, 2012; Silveira‐Moriyama et al., 2018; Termsarasab et al., 2014; Weber, Kohler, Hahn, Neubauer, & Muller, 2013). 16p11.2 microdeletion syndrome is a kind of congenital gene deletion disease, with the clinical manifestations as autism, developmental delay, mental retardation, spinal deformity, and a series of neuropsychiatric developmental diseases (Al‐Jawahiri, Jones, & Milne, 2019; Castelein, Steyaert, Peeters, & van Buggenhout, 2019; Hinkley et al., 2019; Li et al., 2018a; Siu et al., 2019). Phenotypic heterogeneity between patients is obvious, and its pathogenic mechanism is not yet clear.

Therefore, in this study, we conducted whole exome sequencing and copy number variation sequencing (CNV‐seq) on 492 epilepsy children who had benign or refractory epilepsy, in order to find new epileptic phenotypes related to *PRRT2* and verify the positive prediction results of *PRRT2*‐related CNV by qPCR, so as to further clarify the role of 16p11.2 deletion and further understand the clinical and mutational features of *PRRT2*‐related epilepsy.

## 2 METHODS

### 2.1 Subjects

We analyzed 492 epilepsy children with the onset age of 0–14 years between 2016 and 2019 in the Pediatrics Department of Qilu Hospital Affiliated to Shandong University and Linyi people's Hospital Affiliated to Shandong University, China. Their clinical information was retrospectively collected and followed up, including seizure types, onset age, treatment process, growth and development history, previous disease history, family history, intellectual test, cranial magnetic resonance imaging (MRI), and video‐EEG, antiepileptic drugs (AEDs), and age of epilepsy remission. The patients were followed up by phone or visit clinic every three months. Exclusion criteria included seizures caused by nongenetic factors, such as an acquired brain injury, metabolic disease, and clinically phenotypically defined monogenic diseases (e.g., tuberous sclerosis complex). The study protocol was approved by the ethical committee of the Qilu Hospital Affiliated to Shandong University (No. 2016(027)) and Linyi people's hospital Affiliated to Shandong University (No.13003). All guardians signed informed consent forms.

### 2.2 Next‐generation sequencing (NGS) and DNA sequence analysis

Informed consent and blood samples were obtained from all the participants in the families. Genomic DNA was extracted using QIAamp DNA Blood Mini Kit (Qiagen), according to the manufacturer's protocol. Each DNA sample is quantified by agarose gel electrophoresis and Nanodrop 2000 (Thermo). Libraries were prepared using Illumina standard protocol. The amplified DNA was captured with whole exome sequencing. The capture experiment was conducted according to manufacturer's protocol. The junction sequences were trimmed, and the contamination or low‐quality reads were filtered for the raw data. Then, the clean data were aligned to the human reference genome sequence (hg19) by Burrows–Wheeler Alignment. Single nucleotide variation (SNV) and insertion deletion mutation (InDel) were called by Genome Analysis Toolkit. Then, all SNVs and InDels were annotated by ANNOVAR （RRID: SCR_012821）. The mutation sites with frequencies less than 0.05 in the normal population database were screened out, including the 1,000 genome project, Exome Variant Server, and Exome Aggregation Consortium. Mutations were predicted by Mutation Taster (MT), Sorting Intolerant From Tolerant (SIFT, RRID: SCR_012813), PolyPhen‐2 (PP2, RRID: SCR_013189), Genomic Evolutionary Rate Profiling (GERP++, RRID: SCR_000563), and Clustal‐W (RRID: SCR_017277). The selected mutation sites were verified by Sanger sequencing. The analysis of deletions or duplications was performed using low‐coverage massively parallel CNV sequencing (CNV‐seq). After sequencing, the raw data were saved as a FASTQ format, then followed the bioinformatics analysis: First, Illumina sequencing adapters and low‐quality reads (<80bp) were filtered by Cutadapt (1.16) software (RRID: SCR_011841). After quality control, the clean reads were mapped to the UCSC hg19 human reference genome using BWA (0.7.12) software (RRID: SCR_010910). Only uniquely mapped reads were selected. Then, we use GATK (4.0.8.1) (RRID: SCR_001876) Mark Duplicates to remove duplicated reads. Mapped reads were classified into adjustable sliding windows, which were 50 kb in length with 5 kb increments. The coverage of each window was calculated by the read amount and underwent two‐step bias correction (GC correction and population‐scale normalization). After correction, we use the binary segmentation algorithm to localize the segment breakpoints to identify the candidate CNV regions and determination CNV genotype. Then, we use U test and Parallelism test to estimate the genotype and significance of each segment. All the obtained suspected missing repetitive regions were compared with OMIM (RRID: SCR_006437), GeneReviews (RRID: SCR_006560), Decipher (RRID: SCR_006552), ClinVar (RRID: SCR_006169), Database of Genomic Variants (DGV, RRID: SCR_007000) and other databases. CNV‐related genes will also be searched in the Human Phenotype Ontology (HPO, RRID: SCR_006016) database to match similar phenotypes. After the analysis, the data were analyzed for advanced manual analysis, and the suspicious mutation fragments that were highly similar to the clinical phenotype of the proband were selected. Then, real‐time quantitative PCR detecting system (qPCR) experiment was conducted to verify this section, so as to exclude false positive of second‐generation sequencing and ensure the accuracy of the results.

## 3 RESULTS

### 3.1 Genetic analyses

**Figure 1:**
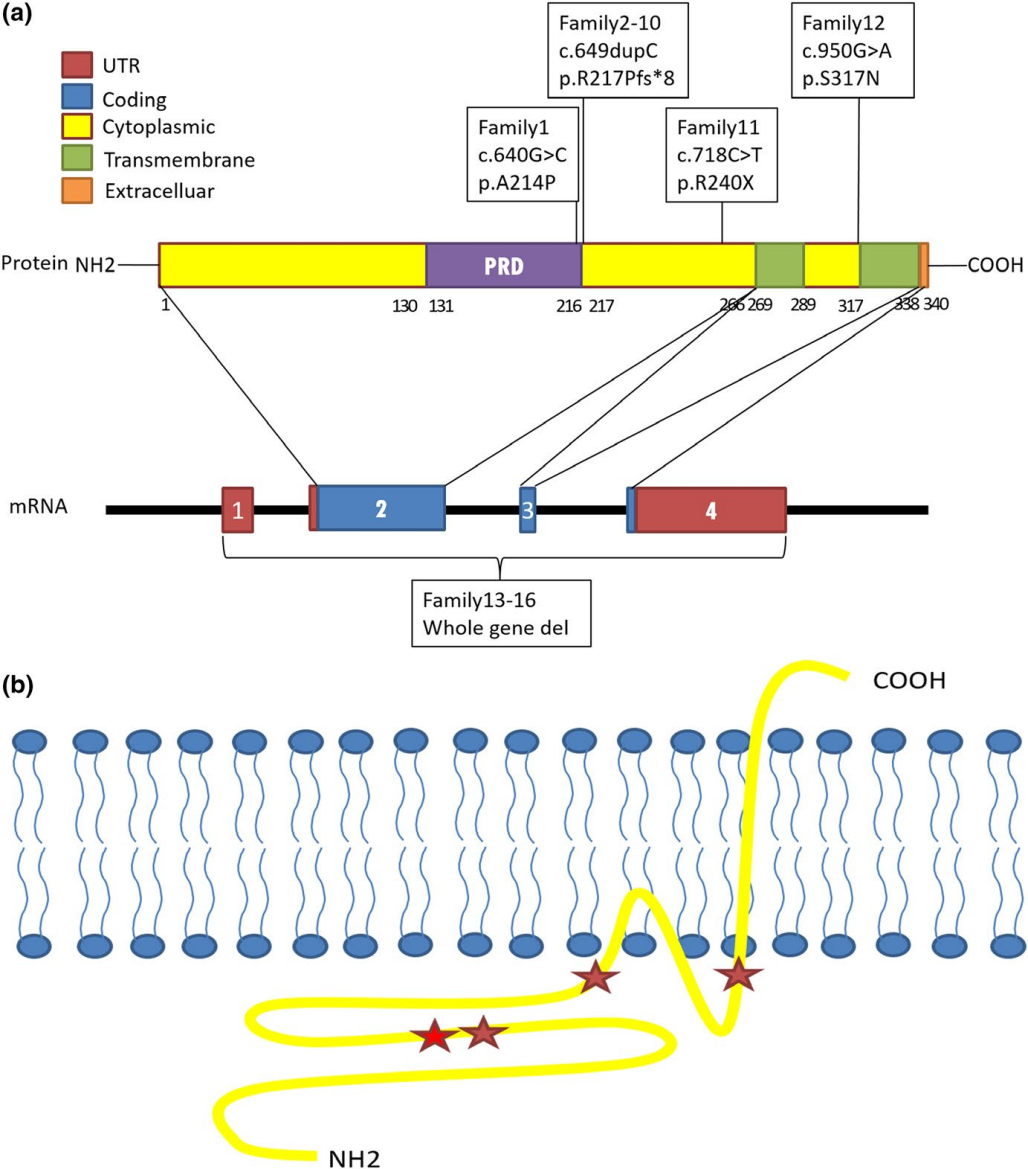
(a) Schematic diagram of the mutations identified in the *PRRT2* gene. PRD, Proline‐rich domain. (b) The mutations identified in Membrane topology of PRRT2. Red star: nine c.649G > C, gray red: the other point mutations

**Figure 2:**
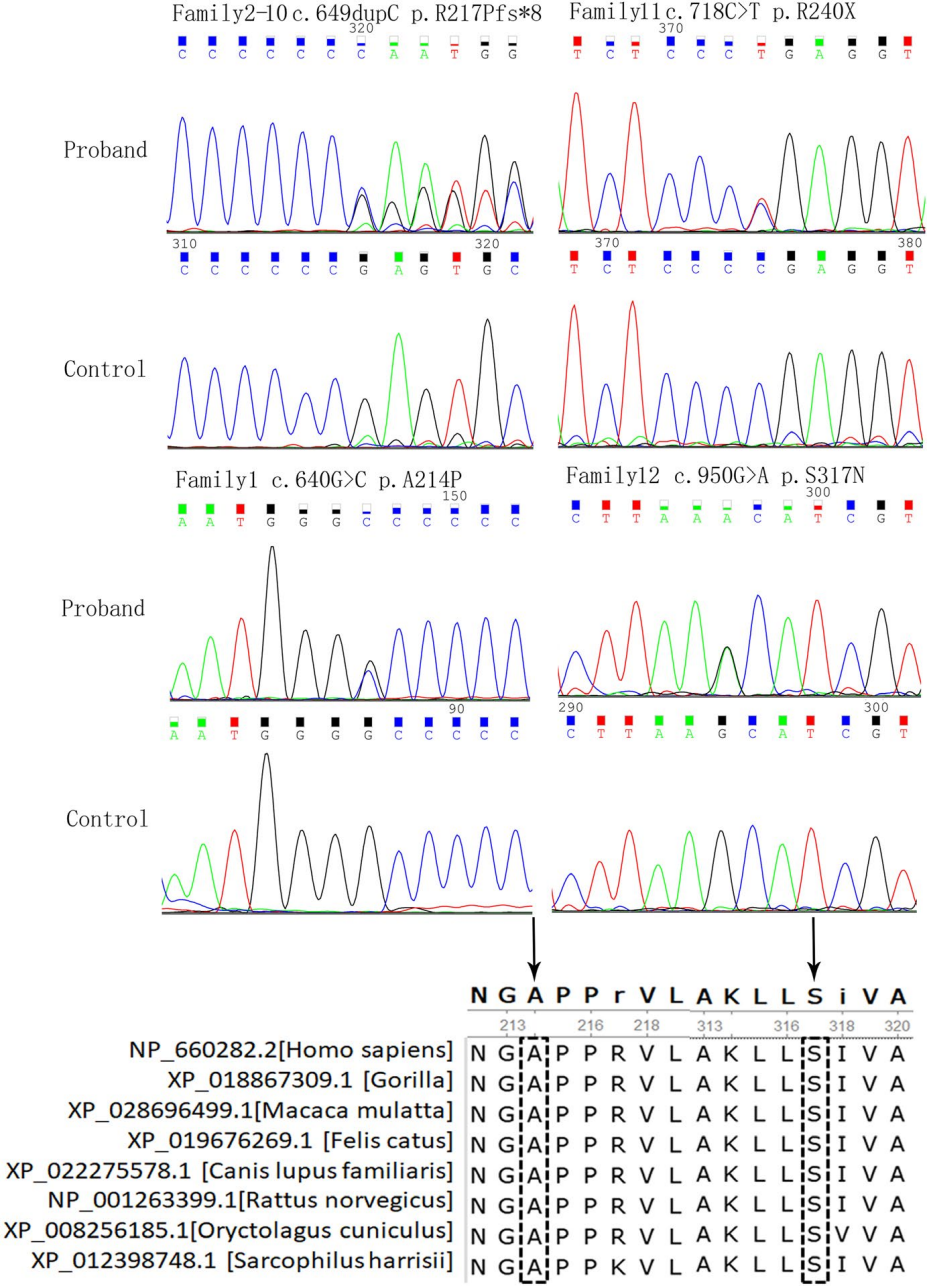
Sequence chromatograms and conversation of amino acid residues affected by the missense mutations. Sequence chromatograms of a *PRRT2* mutation as detected in an affected proband is shown for each family. The black arrow upon orthologous and paralogous protein alignments, showing the high conservation of each amino acid altered by missense mutations in vertebrates and paralogous

**Figure 3:**
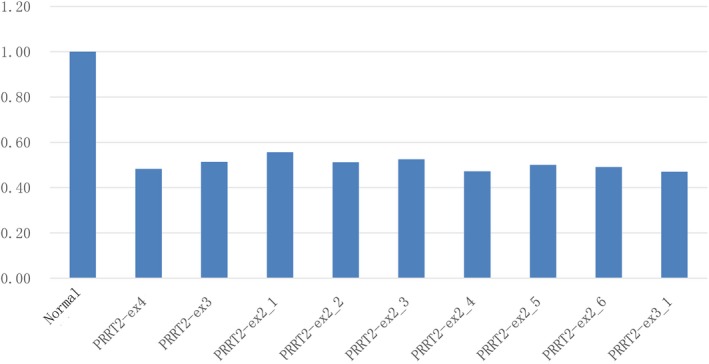
Quantitative PCR validation of whole *PRRT2* gene deletion. Y‐axes represent Log R ratio; the X‐axis indicates the exon on PRRT2

**Figure 4:**
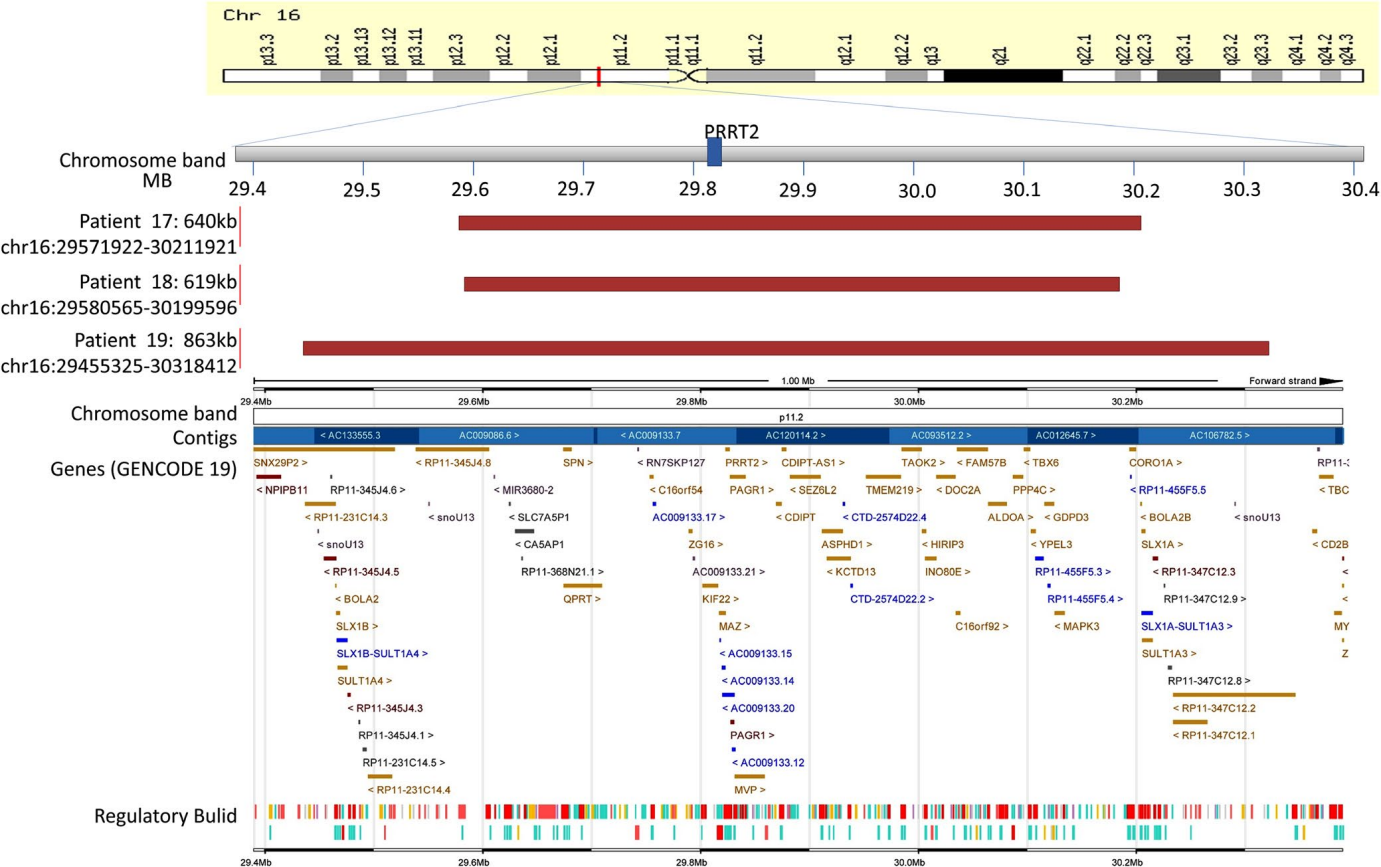
Genomic positions of the deletions and phenotypes of the PKD patients with 16p11.2 deletions. Genomic positions of the 16p11.2 deletions in patients with PKD are shown using red bars

**Figure 5:**
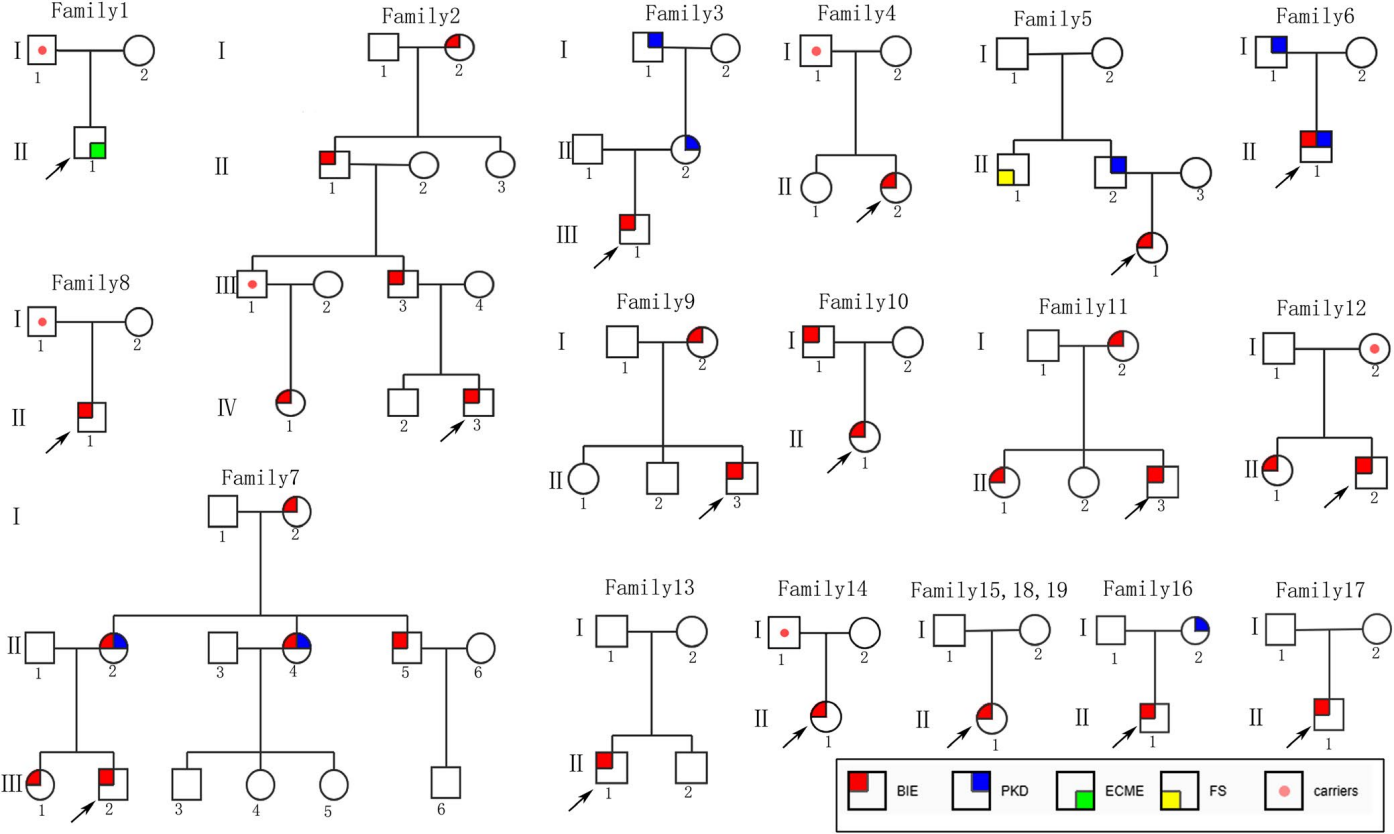
Pedigrees of *PRRT2*‐related epilepsy families. Squares represent males, circles females; Upper left red corner: BIE; Upper right blue corner: PKD; Lower right green corner: ECME; Lower left yellow corner: FS; Dots in the middle of the squares indicate unaffected mutation carriers. The arrows indicate the proband in the family

We screened a cohort of 492 children with epilepsy for mutations in the *PRRT2* gene using whole exome sequencing (WES) and low‐coverage massively parallel CNV sequencing (CNV‐seq) (281 male and 211 female). We found heterozygous *PRRT2*‐related mutations in 19 patients (19/492, 3.86%), six sporadic cases and 13 family cases. Four point mutations were found in 12 patients (12/19, 63.16%), nine of them were c.649dupC mutation (9/12, 75%), four whole gene deletion (4/19, 21.05%) and three 16p11.2 deletions (3/19, 15.79%) (Table 1 and Figure 1). Three mutations were located in exon 2 and one in exon 3. Two missense mutations (c.640G > C/p.Ala214Pro; c.950G > A/p.Ser317Asn), one nonsense mutation (c.718C > T/p.Arg240*), one frameshift mutation (c.649dupC/p.Arg217Profs*8). Missense mutations all affected amino acids of the cytoplasmic domain of proline‐rich transmembrane protein 2 (PRRT2), which are highly conserved in orthologs and in paralogs of PRRT2, and were predicted to be pathogenic by Mutation Taster, Polyphen2, and SIFT (Figure 2, Table 2). Four cases were deletion of whole *PRRT2* gene (Table 1, Figure 1 and Figure 3), three were de novo (3/4, 75%), and all the three 16p11.2 deletions were de novo (3/3, 100%) (Figure 4). Six of the 19 probands for *PRRT2* mutations were de novo (6/19, 31.58%). The inheritance of *PRRT2* mutations in 13 families, family No.2 was four‐generation pedigrees, family No.3,5,7 were three‐generation pedigrees, and the others were two‐generation pedigrees (Figure 5). Five probands inherited the mutation from asymptomatic parents. Six families exhibit incomplete penetrance phenomenon (family No.1: I‐1; family No.2: Ⅲ‐1; family No.4: I‐1; family No.8: I‐1; family No.12: I‐2; family No.14: I‐1).

**Table 1:** Pathogenicity assessment of PRRT2 mutations

Family	Mutation type	Position: Chr 16	Exon	Amino acid changes	Consequence at the protein level	Parents’ analysis	ACMG scoring	ACMG pathogenicity
1	Missense	29,825,015	2	c.640G > C	p.Ala214Pro	Paternal	PS1 + PM2+PP3	LP
2	Frameshift	29,825,024	2	c.649dupC	p.Arg217Profs*8	Paternal	PVS1 + PS1+PM2	P
3	Frameshift	29,825,024	2	c.649dupC	p.Arg217Profs*8	Maternal	PVS1 + PS1+PM2	P
4	Frameshift	29,825,024	2	c.649dupC	p.Arg217Profs*8	Paternal	PVS1 + PS1+PM2	P
5	Frameshift	29,825,024	2	c.649dupC	p.Arg217Profs*8	Paternal	PVS1 + PS1+PM2	P
6	Frameshift	29,825,024	2	c.649dupC	p.Arg217Profs*8	Paternal	PVS1 + PS1+PM2	P
7	Frameshift	29,825,024	2	c.649dupC	p.Arg217Profs*8	Maternal	PVS1 + PS1+PM2	P
8	Frameshift	29,825,024	2	c.649dupC	p.Arg217Profs*8	Paternal	PVS1 + PS1+PM2	P
9	Frameshift	29,825,024	2	c.649dupC	p.Arg217Profs*8	Maternal	PVS1 + PS1+PM2	P
10	Frameshift	29,825,024	2	c.649dupC	p.Arg217Profs*8	Paternal	PVS1 + PS1+PM2	P
11	Nonsense	29,825,093	2	c.718C > T	p.Arg240*	Maternal	PVS1 + PS1+PM2	P
12	Missense	29,825,724	3	c.950G > A	p.Ser317Asn	Maternal	PS1 + PM2+PP3	LP
13	Large deletion			Whole gene del	Absence of protein synthesis	De novo		P
14	Large deletion			Whole gene del	Absence of protein synthesis	Paternal		P
15	Large deletion			Whole gene del	Absence of protein synthesis	De novo		P
16	Large deletion			Whole gene del	Absence of protein synthesis	De novo		P
17	Large deletion	29571922–30211921				De novo		P
18	Large deletion	29580565–30199596				De novo		P
19	Large deletion	29455325–30318412				De novo		P

Abbreviations: P, pathogenic; LP, likely pathogenic.

**Table 2:** Pathogenicity assessment and conservative analysis of 2 missense mutations

Family	Amino acid changes	Consequence at the protein level	Parents’ analysis	SIFT	PolyPhen 2	Mutation Taster	GERP++
1	c.640G > C	p.Ala214Pro	paternal	Damaging	Probably damaging	Polymorphism	3.9 (Conserved)
12	c.950G > A	p.Ser317Asn	maternal	Damaging	Probably damaging	Disease causing	3.75 (Conserved)

### 3.2 Clinical features

The onset age of 492 children ranged from one day after birth to eight years. Thirty‐two (32/492, 6.5%) patients were diagnosed as BIE, 61 as Febrile convulsion plus, 59 as West syndrome, 41 as Dravet syndrome, 12 as Ohtahara syndrome, four as Lennox–Gastaut syndrome, three as West syndrome evolving to Lennox–Gastaut syndrome, four as Doose syndrome, three as childhood absence epilepsy, two as benign childhood epilepsy with central temporal spikes, and one as early childhood myoclonic epilepsy (ECME). Twenty‐nine patients were diagnosed as unclassified epileptic encephalopathy, and 241 patients were diagnosed as unclassified epilepsy. We found *PRRT2*‐related mutations in 19 patients. Among the 19 probands, 10 were males and nine were females. The onset age of the 19 probands ranged from 3 months to 3 years and 2 months. The mode of these 19 patients is six months old, and the median is six months old too. There were 39 patients in these 19 proband families with *PRRT2* mutations. The common clinical features of the 39 patients included one ECME, one febrile seizure (FS), six paroxysmal kinesigenic dyskinesias (PKD), two infantile convulsions with paroxysmal choreoathetosis (ICCA), 12 benign infantile epilepsy (BIE), and 17 benign familial infantile epilepsy (BFIE). The clinical information of 39 patients with *PRRT2* mutations in 19 proband's families is summarized in Table 3.

**Table 3:** The clinical manifestations of the patients with *PRRT2‐*related mutations

Family	Patient	Gender	Age at exam (y.m)	Phenotype	Type of seizures	Seizures in cluster	Onset age of epilepsy (y.m)	Seizure‐free age (y.m)	Onset age of PKD (y.m)	Trigger	Language delay	Brain MRI	Interictal EEG	Onset area of focal seizures	The last follow‐up	
															Present age (y.m)	Current AEDs
1	II1	M	4.7	ECME	GTCS, ME	N	3.2	‐	‐	‐	N	normal	GSW; GPSW	‐	5.3	TPM/VPA/CZP
2	I2	M	‐	BFIE	GTCS	NA	0.6	2	‐	‐	N	NA	normal	‐	‐	N
2	II1	M	60	BFIE	GTCS	Y	0.8	2	‐	‐	N	normal	normal	‐	61	N
2	III3	M	31	BFIE	GTCS Focal	Y	0.3	1.8	‐	‐	N	normal	normal	NA	32	N
2	IV1	F	3.5	BFIE	GTCS Focal	Y	0.3	1.11	‐	‐	N	normal	normal	NA	4.1	N
2	IV3	M	2.1	BFIE	GTCS Focal	Y	1.5	2.1	‐	‐	N	normal	normal	NA	2.9	OXC
3	I1	M	65	PKD	‐		‐	‐	12	SM	N	normal	normal	‐	66	N
3	II2	F	27	PKD	‐	‐	‐	‐	10	SM	N	normal	normal	‐	27	N
3	III1	M	2.1	BIE	Focal	N	0.4	0.8	‐	‐	N	normal	normal	NA	2.8	VPA
4	II2	F	0.6	BIE	Focal	Y	0.5	0.6	‐	‐	N	normal	normal	Right temporal	1.2	OXC
5	II1	M	35	FS	GTCS	N	1	3	‐	‐	N	normal	normal	NA	36	N
5	II2	M	29	PKD	‐	‐	‐	‐	15	SM	N	normal	normal	‐	30	N
5	III1	F	2.9	BIE	Focal	Y	0.5	0.9	‐	‐	N	normal	FD	NA	3.9	VPA
6	I1	M	36	PKD	‐	‐	‐	‐	8	SM	N	normal	normal	‐	38	N
6	II1	M	7.4	ICCA	Focal	N	0.6	1	7	SM	N	normal	normal	NA	8.10	OXC
7	I2	F	72	BFIE	NA	NA	0.6	1	‐	‐	N	NA	NA	NA	73	N
7	II2	F	33	BFIE	Focal	Y	0.6	1	‐	‐	N	NA	NA	NA	34	N
7	II4	F	31	BFIE	Focal	Y	0.5	1.3	‐	‐	N	normal	normal	NA	32	N
7	II5	M	28	ICCA	Focal	Y	0.7	1	6	SM	N	normal	normal	NA	29	N
7	III1	F	8	BFIE	Focal	Y	0.5	1.7	‐	‐	N	normal	normal	NA	9.6	N
7	III2	M	0.5	BFIE	Focal	Y	0.4	1.2	‐	‐	N	normal	normal	NA	1.11	OXC
8	II1	M	2.4	BIE	Focal	Y	0.8	2	‐	‐	N	normal	normal	NA	3.8	OXC
9	I1	F	32	BFIE	Focal	N	0.6	1	‐	‐	N	normal	normal	NA	32	N
9	II1	M	1	BFIE	Focal	N	0.4	0.8	‐	‐	N	normal	normal	NA	1.4	LEV
10	I1	M	25	PKD	‐	‐	‐	‐	17	SM	N	normal	normal	‐	25	CBZ
10	II1	F	0.5	BIE	Focal	Y	0.5	NA	‐	‐	N	normal	FD	Right temporal	0.6	N
11	I22	F	31	BFIE	Focal	N	0.6	1.6	‐	‐	N	normal	normal	NA	32	N
11	II1	F	7	BFIE	Focal	Y	0.4	1	‐	‐	N	normal	normal	NA	8	N
11	II3	M	0.4	BFIE	Focal	Y	0.3	0.6	‐	‐	N	normal	normal	Right frontal	1.2	VPA
12	II1	F	2	BFIE	Focal	N	0.6	0.11	‐	‐	N	normal	normal	NA	2.2	LEV
12	II2	M	0.6	BFIE	Focal	N	0.5	0.6	‐	‐	N	normal	normal	NA	0.8	LEV
13	II1	M	0.8	BIE	Focal	Y	0.5	0.11	‐	‐	N	normal	normal	Left occipital	3.4	LEV
14	II1	F	1.2	BIE	Focal	N	1	1.5	‐	‐	N	normal	normal	NA	4.5	LEV
15	II1	F	2.5	BIE	Focal	N	0.8	1.8	‐	‐	N	normal	normal	NA	3.6	OXC
16	I11	F	25	PKD	‐	‐	‐	‐	18	SM	N	normal	normal	‐	25	N
16	II1	M	0.6	BIE	Focal		0.5	NA	‐	‐	N	normal	FD	Left occipital	0.7	OXC
17	II1	M	0.6	BIE	Focal	Y	0.5	1	‐	‐	N	normal	FD	NA	2.7	VPA
18	II1	F	1.2	BIE	Focal	Y	0.6	1.3	‐	‐	N	normal	normal	NA	1.6	LEV
19	II1	F	1.6	BIE	Focal	Y	0.7	1	‐	‐	mild	normal	FD	NA	1.9	LEV/VPA

Abbreviations: AEDs, antiepileptic drugs; BFIE, benign familial infantile epilepsy; BIE, benign infantile epilepsy; CBZ, carbamazepine; CZP, clonazepam; F, female; FD, focal discharge; Focal, focal motor seizures; FS, febrile seizures; GPSW, generalized polyspike wave; GSW, generalized spike‐wave; GTCS, generalized tonic–clonic seizures; ICCA, infantile convulsions with paroxysmal choreoathetosis; LEV, levetiracetam; M, male; ME, myoclonic seizures; N, none; NA, Not available; OXC, oxcarbazepine; PKD, paroxysmal kinesigenic dyskinesias; SM, sudden movement; VPA, sodium valproate; Y, yes; y.m, years.months.

**Figure 6:**
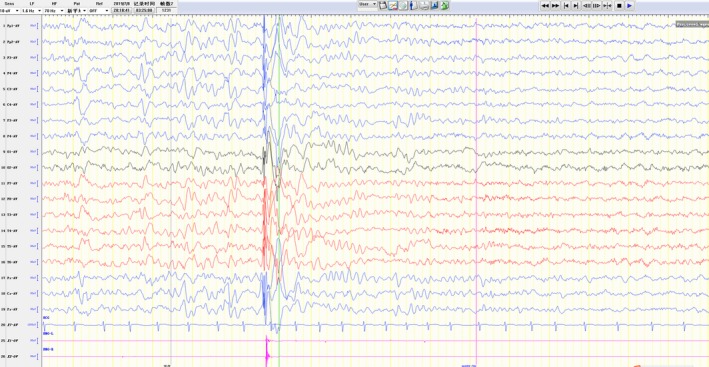
Ictal EEG and EMG tracing of patient II1 in family 1. High to very high 3‐4Hz generalized polyspike waves with time‐locked relation between muscle activation corresponding to a sudden shake of the whole body

The proband with ECME was a boy aged four years and seven months old, the only child of nonconsanguineous parents. The child was born via spontaneous vaginal delivery at 37 weeks gestation, without asphyxia. The patient had normal cranial MRI and developmental quotient, no special family history or personal history, and no regression of development after onset. When the child was three years and two months old, one generalized tonic–clonic seizures (GTCS) occurred without obvious inducement. Six months after that, GTCS occurred when he had a fever. Then, another form of seizure occurred, characterized by a quick shake of the upper limbs or whole body. It occurred several times a day, without other associated symptoms. Interictal electroencephalography (EEG) showed normal background activity and high to very high amplitude spike or polyspike wave discharges at 3–4 Hz. Ictal EEG detected myoclonic seizures as generalized polyspike wave with a time‐locked relation to muscle activation (Figure 6). The whole gene sequencing revealed a heterozygous mutation (c.640G > C), resulting in protein truncation (p.Ala214Pro). Three other variants of likely pathogenic or pathogenic were identified in this patient as well (*VWF*, *SYNGAP1,* and *ABCB4*). *SYNGAP1* (c.3964_3965insCCCCCCC/p.P1326Tfs*38) and *ABCB4* (c.1015dupT/p.S339Ffs*16) variations were not found after one generation verification. *VWF* (c.5014G > A/p.G1672R) mutation originated from the father of the child. However, neither the child nor the father has hemophilia phenotype, which is inconsistent with the clinical manifestations, so it is not considered as a pathogenic gene mutation. The patient was insensitive to valproate sodium (VPA), levetiracetam (LEV), topiramate (TPM), and clonazepam (CZP).

The onset age of the thirty‐one patients with BIE or BFIE ranged from three months to one year and three months, both the mode and median age were six months. Among them, there were 16 patients with onset age of <6 months (16/31, 51.61%), 13 patients with onset of disease at 6–12 months (13/31, 41.94%), and one patient with onset after 12 months (1/31, 3.22%). Remission age ranged from six months to two years and one month. The type of seizures in one patient was unknown, two patients only had generalized tonic–clonic seizures (GTCS) (2/30, 6.67%), 25 patients had focal motor seizures (25/30, 83.33%), three patients had both types of seizures (10%), and 20 patients had seizure clusters (20/29, 68.97%). All patients had normal intelligence, except one patient with 863kb deletions of 16p11.2 who had a mild language delay. All the PKD patients were triggered by sudden movement. All patients had a normal brain MRI. Only one patient had generalized polyspike wave, and five patients had focal transient discharges in interictal EEG. Focal seizures originating in the frontal region were recorded in one patient, two from the temporal region, and two from the occipital region. Most patients were treated effectively with sodium valproate (VPA) or oxcarbazepine (OXC); the child with myoclonic seizures was not sensitive to antiepileptic drugs.

## 4 DISCUSSION

*PRRT2* is localized to chromosome 16p11. 2, with a total length of 3,794 bp. It contains four exons and codes 340 amino acids and is highly expressed in the cerebral cortex, basal ganglia, and cerebellum. *PRRT2* is composed of n‐terminal sequence rich in proline (n‐glycosylation site), two transmembrane domains and c‐terminal sequence (Rossi et al., 2016), and its transmembrane region is highly conserved, suggesting important physiological functions. Studies have shown that PRRT2 has analogues in vertebrates such as humans, gorillas, macaques, and mice, while no homologous products have been found in invertebrates such as nematodes. In humans and rodents, PRRT2 is a neural protein and is most expressed in the cerebellum, basal ganglia, and neocortex. PRRT2 serves as a regulator of the SNARE complex and provides a circuit mechanism underlying the *PRRT2*‐related diseases (Tan et al., 2018). The mutant PRRT2 may affect glutamate signal transduction and glutamate receptor activity through its weak interaction with synaptic proteins SNAP‐25, leading to an increase in glutamate release, which in turn leads to overexcitation of neurons (M. Li et al., 2015). PRRT2 is closely related to Ca^2+^ sensing mechanisms and plays an important role in the final phase of neurotransmitter release by interacting with SNAP‐25 and synaptotagmin (Tan et al., 2018; Valente et al., 2016).

Currently, the clinical phenotypes caused by *PRRT2* gene mutation are mainly BFIE, PKD, and ICCA. Other rare phenotypes include hemiplegic migraine (HM) and sporadic benign infant epilepsy (BIE), suggesting phenotypic heterogeneity in *PRRT2* mutations. So far, most of the mutations associated with the *PRRT2* have been labeled "benign." However, west syndrome and FS were identified in some *PRRT2* families (Djemie et al., 2014; Igarashi et al., 2016), suggesting that the spectrum of *PRRT2*‐related diseases may be broader. To explore the phenotypic boundaries of *PPRT2*‐related mutations, we screened a wide range of benign and severe infantile epilepsy patients. In this study, we found one ECME, one FS, six PKD, two ICCA, 12 BIE, and 17 BFIE in 39 patients of 19 probands’ families. This is the first time *PRRT2* mutation has been discovered in ECME. The proband with the onset age of three years and two months had myoclonic seizures and GTCS, and febrile or afebrile GTCS appeared before myoclonic seizures, normal cranial MRI and developmental quotient, 3–4 Hz generalized spike‐wave, and polyspike wave on interictal EEG. According to the characteristics of ECME summarized by Yang et al. (2017)), we diagnosed the child as ECME. This is the first report of *PRRT2* mutation in ECME and extends the spectrum of diseases associated with PRRT2.

In our study, five probands of BIE or BFIE families had their first seizure episodes caused by diarrhea (family 5, 8, 11, 12, 16), and some of them had clusters of seizures. We could not figure out whether these patients manifested convulsions with mild gastroenteritis (CwG) or the symptoms were the presentation of BFIE itself at first. CwG is characterized by the following clinical features: infants aged 6 months to 3 years having afebrile generalized convulsions induced by mild gastroenteritis; clustering seizures; normal laboratory examination results including electrolytes, blood glucose and cerebrospinal fluid; normal interictal EEG and brain MRI; and good prognosis. As the disease progressed, the children developed unprovoked seizures a few months later that set them apart from the CwG. Moreover, *PRRT2* mutations have not been reported in such patients with CwG, which is one of the distinguishing points with *PRRT2*‐related diseases (Ishii et al., 2013). Therefore, when we encounter the first cluster or single seizure induced by mild gastroenteritis in clinical, we should pay attention to the family history and the follow‐up prognosis. The patient with family history could chose genetic test to assist diagnosis and prognosis.

The reported mutation types summarized by Darius (Ebrahimi‐Fakhari, Saffari, Westenberger, & Klein, 2015) include missense, nonsense, insertion or deletion of bases, splicing, deletion of fragments or whole genes, and deletion of other adjacent genes. In our study, 63.16% patients had reported point mutations inherited from symptomatic or asymptomatic parents, two missense mutations (Heron et al., 2012; Liu et al., 2016), one nonsense (Lee et al., 2012), one frameshift mutation (Chen et al., 2011), and c.649dupC mutation is also the hot spot mutation. Since the majority of *PRRT2* mutations are truncating mutations that lead to loss of function or haploinsufficiency of PRRT2, copy number deletions of this gene are suspected of causing *PRRT2‐*related diseases. There were still 21.05% of patients who had whole gene deletion, and 15.79% had 16p11.2 deletions, most of them were de novo. On the contrary, all the point mutations in this study were inherited. It indicates that screening for *PRRT2* CNVs is necessary in these patients, especially in sporadic cases. Only one patient with the whole gene deletion inherited from asymptomatic father, *PRRT2* point mutations had incomplete extraneous dominance in previous studies, but the whole gene heterozygous deletion had no clinical phenotype, which has not been reported. Chromosome 16p11.2 deletion syndrome is a genetic disorder associated with multiple system abnormalities, including intellectual impairment, language developmental disorders, seizures, psychical and psychological disease, PKD, obesity, hearing loss, and cardiac defects (Li et al., 2018b; Yang et al., 2015). In our study, all three BIE patients with 16p11.2 deletion had focal motor seizures, only one with the largest 16p11.2 deletion of 863kb had mild language delay, which indicates that pure BIE or with mild language delay can be a phenotype of 16p11.2 deletion syndrome that extending the phenotype of 16p11.2 deletions to typical BIE/BFIE. Screening for 16p11.2 deletions should be prescribed for patients with BIE, particularly in sporadic cases, although it is not common. A case of BIE and normal neurodevelopment in a child with a loss of 1.064 MB 16p11.2 was reported (Milone, Valetto, Bertini, & Sicca, 2017), with regression leading to autism, intelligence retardation, and language impairment at 18 months of age. We had not found the autism or intellect regression in the other two children with 16p11.2 deletions of our study who had excellent seizure‐free and developmental outcomes as followed up at 18 months to 31 months old. But, the patients with 16p11.2 deletions still need long‐term clinical follow‐up.

## 5 CONCLUSIONS

In conclusion, *PRRT2* mutations can be inherited or de novo. *PRRT2* mutation‐related epilepsy has incomplete penetration rate and phenotypic heterogeneity. The clinical spectrum of *PRRT2* mutation includes BIE, BFIE, ICCA, PKD, FS, and ECME. The *PRRT2*‐related mutations contained point mutations, whole gene deletion, and 16p11.2 deletions. Point mutations were mainly inherited, and large microdeletion mutations were mostly de novo. Screening for 16p11.2 deletions should be prescribed for patients with BIE, particularly in sporadic cases. Our report expands the mutation and clinical spectrum of *PRRT2*‐related epilepsy.

## CONFLICT OF INTEREST

All authors declare that there is no conflict of interest.

## AUTHOR CONTRIBUTION

LY and BML were responsible for the original concept and the overall design of the research. LY, SYQ, XFY, YFL, LYX, JLY, and BML analyzed the EEG results and diagnosed patients. XL, LY, NX, DQZ, and YN collected the clinical data and sample. LY, CPY, and FL carried the experiments and analyzed the sequencing data. LY, CPY, JLY, and BML wrote and revised the manuscript. All authors read and approved the final manuscript.

## Data Availability

The data that support the findings of this study are available on request from the corresponding author. The data are not publicly available due to privacy or ethical restrictions.
